# Effect of Different Initial CaO/SiO_2_ Molar Ratios and Curing Times on the Preparation and Formation Mechanism of Calcium Silicate Hydrate

**DOI:** 10.3390/ma16020717

**Published:** 2023-01-11

**Authors:** Jianfang Wu, Hongqiang Liao, Zhuohui Ma, Huiping Song, Fangqin Cheng

**Affiliations:** 1 State Environment Protection Key Laboratory of Efficient Utilization Technology of Coal Waste Resources, Institute of Resources and Environmental Engineering, Shanxi University, Taiyuan 030006, China; 2 Shanxi Pingshuo Gangue-Fired Power Generation Co., Ltd., Shuozhou 036800, China

**Keywords:** C-S-H formation mechanism, pozzolanic reaction, silica fume, calcium oxide, C/S molar ratio, ^29^Si MAS-NMR, curing times

## Abstract

To better understand the pozzolanic activity in fly ash used as a supplementary cementitious material in cement or concrete, calcium silicate hydrate (C-S-H) has been synthesized by adding silica fume to a supersaturated calcium hydroxide solution prepared by mixing calcium oxide and ultrapure water. Thermogravimetric analysis results have revealed the variation in the weight loss due to C-S-H in the samples and the conversion ratio of calcium oxide (the *μ_CaO_* value), which represents the proportion of calcium oxide in the initial reaction mixture used to produce C-S-H, with curing time. The weight loss due to C-S-H and the *μ_CaO_* value were both maximized (13.5% and 90.4%, respectively) when the initial C/S molar ratio was 1.0 and the curing time was 90 d. X-ray diffraction (XRD) analysis has indicated that C-S-H in the samples after curing for 7 d had the composition Ca_1.5_SiO_3.5_·xH_2_O. ^29^Si magic angle spinning (MAS) nuclear magnetic resonance (NMR) analysis has revealed that the degree of polymerization of C-S-H increased with an increase in curing time for samples with an initial C/S molar ratio of 1.0. The ratio of internal to terminal tetrahedra (Q^2^/Q^1^) increased from 2.29 to 4.28 with the increase in curing time from 7 d to 90 d. At curing times ≥ 28 d, a leaf-like C-S-H structure was observed by scanning electron microscopy (SEM). An ectopic nucleation–polymerization reaction process is proposed for the formation mechanism of C-S-H.

## 1. Introduction

Calcium silicate hydrate (C-S-H) is the main hydration product in Portland cement; it makes up about 50% of the hardened paste volume and plays an essential role in controlling its engineering properties [1,2]. Herein, we use the standard abbreviations C = CaO, S = SiO_2_, and H = H_2_O, as generally employed in cement chemistry [3]. Understanding the composition and structure of C-S-H is important for adjusting and controlling the mechanical properties and stability of hardened cement and concrete [2]. The compressive strength of cement-based materials is positively correlated with the content of C-S-H, both of which increase with an increase in the curing time (≤ 28 d) [4,5]. Thus, the inherent compressive strength of cementitious materials may be improved by incorporating C-S-H, which may be generated in situ or deployed as an additive [6,7,8]. There are two main experimental approaches for studying C-S-H [9], namely, characterizing it within a cement paste [10] and its synthesis. The synthesis methods mainly include chemical precipitation, solution reaction, and hydrothermal synthesis, such as using Si(OH)_4_ and CaCl_2_ solutions under alkaline conditions, Ca(NO_3_)_2_ and Na_2_SiO_3_ solutions, or NaOH, CaCO_3_, and SiO_2_ as raw materials [11,12,13]. C-S-H is made up of nanocrystalline regions with an atomic structure resembling that of tobermorite and/or jennite in both pastes and synthetic systems [14]. By studying the effect of adding different contents of silica fume on the composition of C-S-H in cement paste, Rossen et al. found that the microstructure development of cement–silica fume blends is very different from that in plain cement and portlandite (CH) tends to precipitate as platelets and even around clinker grains as “CH rims” and is then consumed [9]. Maddalena et al. observed that the final composition of C-S-H depends only on the initial C/S ratio and that the silica particle size affects the rate of reaction [3]. In recent years, molecular dynamics simulation has been used to study the basic structure and mechanical properties of C-S-H at the nanoscale. Hou et al. [15] found that with an increasing C/S ratio, the silicate chain length gradually decreases, and more defective silicate chains appear, which could weaken the mechanical performance of C-S-H. Izadifar et al. [16,17] studied the correlation between the composition and mechanical properties of C-S-H and the role of interlayer water by infrared spectroscopy and density functional theory (DFT). Abdolhosseini et al. [18] proposed a combinatorial method for optimizing the properties of cement hydrates. However, the formation mechanism of C-S-H is still unclear.

C-S-H is also the product of the volcanic reaction, which refers to the reaction between hydrated lime and active silica contained in siliceous materials [10]. The common pozzolanic reaction occurs in the hydration of cement incorporating industrial solid wastes such as fly ash and blast furnace slag. It is for this reason that fly ash may be used as a supplementary cementitious material to partially replace cement clinker or as a mineral component to be admixed with cement or concrete [19,20,21]. This may also reduce the CO_2_ emissions of the cement industry, which currently account for 8% of global CO_2_ emissions, and this value may continue to rise due to the demand for cement with the realization of infrastructure projects [22]. Meanwhile, it could improve the utilization of fly ash, which is currently only 25% of that generated globally [23]. However, the composition of fly ash is too complex and variable for it to be used reproducibly. According to research reports, 316 discrete mineral components and 188 complex mineral phases have been detected in fly ash [19,20,24]. Due to the complex composition of fly ash, its properties are highly variable. Its composition is mainly affected by the type of coal, the combustion conditions, as well as the conditions of capture and storage. The complexity and variability of fly ash components make it difficult to accurately control the properties of fly-ash-based secondary products, which has greatly limited the large-scale commercial application of fly ash. The amount of C-S-H in fly-ash-based cementitious materials is related to material properties. Therefore, it is necessary to understand the formation mechanism of C-S-H by pozzolanic reaction in order to identify ways of controlling the chemical composition of fly ash. Because silica fume is an amorphous silica, its reaction with Ca(OH)_2_ can closely simulate the pozzolanic reaction between fly ash and Ca(OH)_2_ [25]. Thus, the study of C-S-H prepared from silica fume, calcium oxide, and ultrapure water should enhance our understanding of the pozzolanic reaction in fly-ash-based cementitious materials.

In the present work, C-S-H has been prepared from silica fume, calcium oxide, and ultrapure water with different initial C/S molar ratios and curing times at room temperature in order to simulate the hydration of fly-ash-based cementitious materials. The influences of the initial C/S molar ratio and curing time on the formation mechanism of C-S-H have been explored. It is hoped that this work will lay a foundation for the optimization of the component design of fly-ash-based cementitious materials through stoichiometry and phase composition, thereby eliminating the effect of variable compositions on the utilization of fly ash and expediting the surmounting of peak carbon and the realization of carbon neutrality in the cement industry.

## 2. Experimental Section

### 2.1. Raw Materials

**Figure 1:**
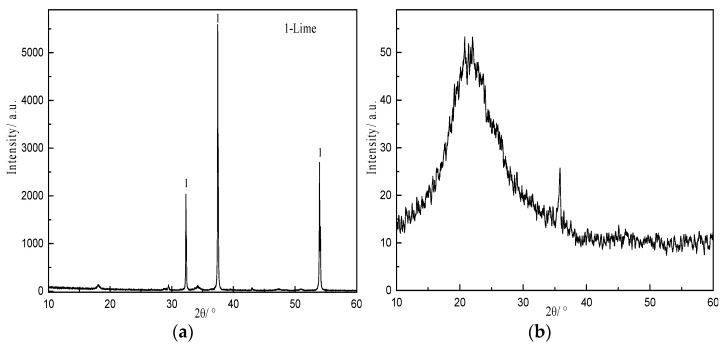
XRD patterns of calcium oxide (**a**) and silica fume (**b**).

**Figure 2:**
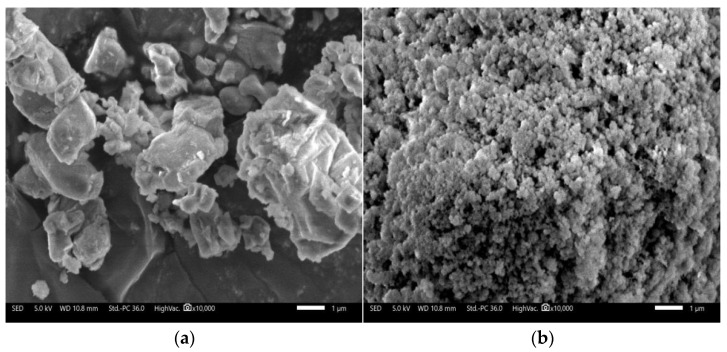
SEM images of calcium oxide (**a**) and silica fume (**b**).

C-S-H phases were synthesized from calcium oxide (AR, CaO content ≥ 98%, Sinopharm Chemical Reagent Co., Ltd., Zhengzhou, China), silica fume (industrial grade, chemical composition shown in Table 1, A Material Company, Henan, China), and ultrapure water (pH 6.7, conductivity < 0.2 μS/cm, total organic carbon (TOC) 22 ppm, and resistivity ≤ 18 MΩ). The X-ray diffraction (XRD) pattern (Figure 1) showed that the major mineral composition of the calcium oxide was lime (PDF#37-1497). The XRD pattern of the silica fume showed only broad, diffuse features, implying that the sample was essentially amorphous. Scanning electron microscopy (SEM) observation revealed that the silica fume particles were mostly spherical and no more than 1 μm in diameter, whereas the calcium oxide particles were larger and irregular, as seen in Figure 2.

### 2.2. Synthesis

**Figure 3:**
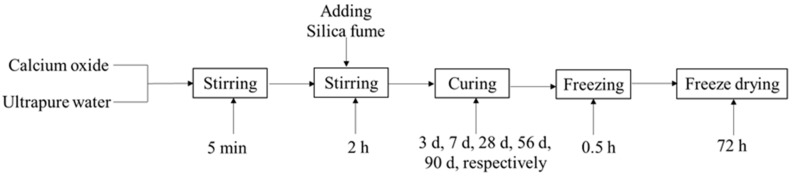
Experimental scheme.

The experimental scheme is shown in Figure 3. First, ultrapure water (22.40 mL) was placed in a 100 mL beaker. Calcium oxide (2.8571 g) was then added with magnetic stirring. After 5 min, a certain amount of silica fume was quickly added, and the mixture was stirred for 2 h. Thereafter, the beaker was sealed with polyethylene film to prevent carbonation of hydrated calcium silicate in the sample, and the mixture was set aside at room temperature for curing for 3 d, 7 d, 28 d, 56 d, or 90 d. After the designated curing time, the beaker was cooled at –18 °C for 30 min to freeze the sample for subsequent freeze-drying. Finally, several holes were made in the sealing film, and the contents of the beaker were freeze-dried for 72 h in order to retain the micro-morphology of the product. The amount of silica fume added was in accordance with the required initial C/S molar ratio, 0.5, 1.0, 2.0, or 2.5.

### 2.3. Analysis

The freeze-dried samples were analyzed by thermogravimetric analysis (TGA), X-ray diffractometry (XRD), and scanning electron microscopy (SEM).

TGA data were acquired with a PerkinElmer Pyris 1 apparatus (PerkinElmer, Waltham, MA, USA), the accuracy of which was better than 0.02%. Weight losses from the samples, which were used to measure the production of C-S-H, were recorded between 50 °C and 800 °C, heating at a rate of 10 °C/min under a high-purity nitrogen atmosphere. The amounts of substances in the samples were quantified from the weight losses by the tangential method [26]. The weight loss was obtained as the difference in weight at the intersections of a tangent drawn at the point of maximum slope in the weight loss curve and straight lines fitted to the data above and below the decomposition point [3].

XRD patterns were recorded on a D2PHASER (Bruker) employing Cu-K_α_ radiation at a scanning rate of 5.6°/min in the range from 10° to 60°, with generator settings of 30 kV/10 mA, to determine the sample composition. The functions “Find Peaks” and “Peak Search Report” in Jade 5.0 were used to determine the characteristic peaks in the pattern and to determine the height and area of each characteristic peak.

For microstructural characterization, the samples were coated with platinum and observed by means of a JSM-670F microscope (JEOL, Tokyo, Janpan) operated at an accelerating voltage of 5 kV under a high vacuum, which was coupled to an energy-dispersive spectroscopy (EDS) analyzer.

^29^Si nuclear magnetic resonance (NMR) spectra were recorded on an Agilent 600 DD2 spectrometer (Agilent, Palo Alto, California, USA, magnetic field strength 14.1 T) at a resonance frequency of 199.13 MHz for ^29^Si under magic-angle spinning (MAS) conditions. The powder samples were placed in a pencil-type zirconia rotor of length 4.0 mm. The spectra were obtained at a spinning frequency of 8 kHz (4 μs 90° pulses), with a cycle delay of 3 s. The Si signal of tetramethylsilane (TMS) at 0 ppm was used as a reference for ^29^Si chemical shifts. The number of scans was 1024. The observed ^29^Si resonances were analyzed using the Q^n^ classification, where *n* (0–4) represents the number of bridging oxygen atoms connecting each tetrahedron silica unit with other Si atoms [27].

## 3. Results and Discussion

### 3.1. Thermogravimetric Analysis

**Figure 4:**
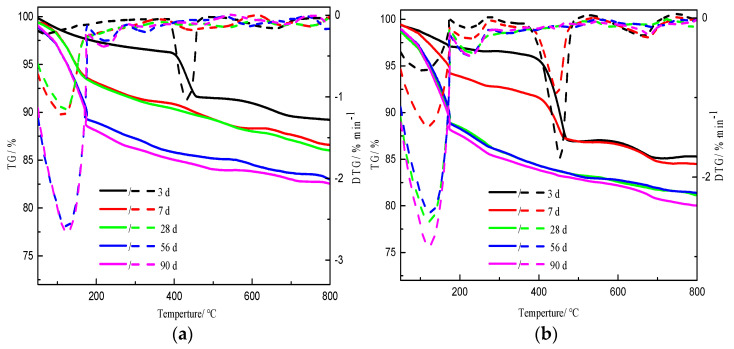

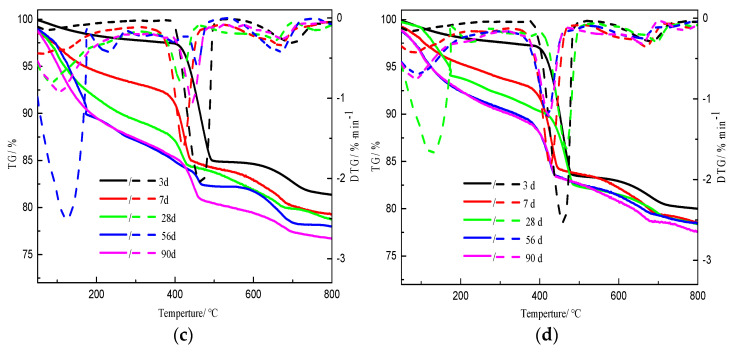
TG/DTG traces of samples: (**a**) initial C/S molar ratio 0.5; (**b**) initial C/S molar ratio 1.0; (**c**) initial C/S molar ratio 2.0; and (**d**) initial C/S molar ratio 2.5. The solid lines represent the TG results of the samples, while the dotted lines represent the corresponding DTG results.

TG/DTG traces of prepared samples cured for different durations with different initial C/S molar ratios are shown in Figure 4. Multi-step weight loss can be seen in each trace. The first weight loss step in the range from 50 °C to 300 °C corresponds to the loss of loosely bound interlayer water [28,29] and the typical dehydration of C-S-H [30,31]. The second weight loss step in the range of 300–550 °C corresponds to the dehydration of calcium hydroxide (C-H) [3]. The final weight loss step between 550 °C and 750 °C corresponds to the decarbonization of calcium carbonate (CaCO_3_) [32], formed during the preparation and curing processes.

For an initial C/S molar ratio of 0.5, as the curing period was extended, the weight loss due to C-S-H increased, the weight loss due to calcium hydroxide decreased, while the weight loss due to calcium carbonate did not vary much, as shown in Figure 4a. This was essentially consistent with the previous findings, which showed that the C-H content decreased with increasing curing time at constant reaction temperature with the same amount of silica fume [4,9]. There was little difference in the weight losses due to calcium hydroxide for samples cured for 28 d, 56 d, and 90 d. However, after curing times of 56 d and 90 d, the weight loss due to C-S-H was obviously higher than that of the sample cured for 28 d.

When the initial C/S molar ratios were 1.0, 2.0, and 2.5, the changes in weight losses due to calcium hydroxide and C-S-H with increasing curing time, were essentially the same as those at a C/S ratio of 0.5, as shown in Figure 4b–d.

**Figure 5:**
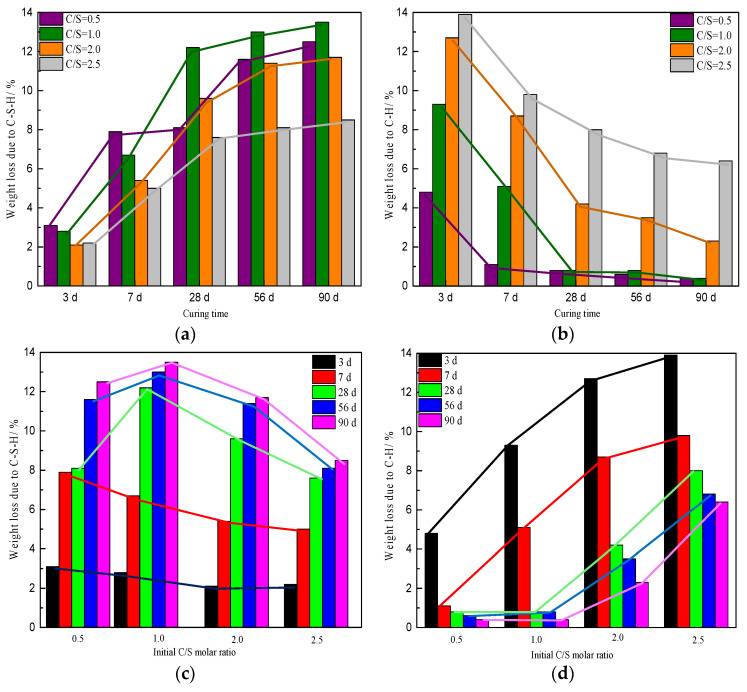
Weight losses due to (**a**,**c**) C-S-H and (**b**,**d**) C−H of samples cured for different times and with different initial C/S molar ratios.

For the samples with an initial C/S molar ratio of 1.0, the weight loss due to calcium hydroxide did not vary after curing times of 28 d, 56 d, and 90 d, but the weight loss due to C-S-H was obviously higher than that of the sample cured for 7 d, in contrast to the situation when the initial C/S molar ratios were 0.5 and 2.5. There were still significant weight losses due to calcium hydroxide from the samples with initial C/S ratios of 2.0 and 2.5 when the curing time was extended to 90 d. All of the weight losses from the respective samples shown in Figure 4 were calculated by the above-described method. The results are shown in Table 2 and Figure 5.

From Table 2 and Figure 5a, it is clear that the weight loss due to C-S-H from the sample with an initial C/S molar ratio of 0.5 was the highest and that that from the sample with a C/S molar ratio of 1.0 was the second highest when the curing time was ≤ 7 d. Losses from the samples with C/S molar ratios of 2.0 and 2.5 showed little difference after a curing time of 3 d or 7 d. This indicated that in the early stage (curing time ≤ 7 d), the greater the silica fume content in the reactants, the more water in the C-S-H of the product. In other words, a high silicon content promoted the formation of C-S-H in the early stage of curing. The weight loss due to C-S-H first increased and then decreased with an increase in the initial C/S molar ratio after curing times of 28 d, 56 d, and 90 d, reaching a relative maximum when the initial C/S molar ratio was 1.0. The maximum weight loss due to C-S-H was 13.5% for the sample cured for 90 d with an initial C/S ratio of 1.0. The weight loss due to C-H essentially increased with increasing initial C/S molar ratio after each curing time, as shown in Figure 5b.

The weight loss due to C-S-H gradually increased with the extension of curing time for samples with the same initial C/S molar ratio, whereas the weight loss due to C-H decreased or remained essentially unchanged, as shown in Table 1 and Figure 5c,d. An increase in curing time was conducive to an increase in the water content of C-S-H. When the initial C/S molar ratios were 2.0 and 2.5, the weight losses due to C-H were significantly higher than those from the other samples after the same curing time (≥28 d).

However, the total masses of each sample were not the same because of their different initial C/S molar ratios. Thus, the weight loss due to C-S-H could not be directly used to accurately characterize the C-S-H content in each sample. Therefore, further analysis of C-S-H production was needed.

### 3.2. Formation of C-S-H

In order to compare the effects of different initial C/S molar ratios on the generation of C-S-H, the parameter *μ_CaO_* (%) is defined as the conversion ratio of calcium oxide to represent the proportion of calcium oxide in the initial reaction mixture. The calculation formula is as follows:

(1) $$\mu_{CaO}=\frac{n_{I}-n_{C-H}-n_{CaCO_{3}}}{n_{I}}\times 100$$

where $n_{I}$ is the molar percentage of effective calcium oxide in the initial reactants, and $n_{C-H}$ and $n_{CaCO_{3}}$ are the molar percentages of calcium hydroxide and calcium carbonate in the product, respectively.

In this study, the total mass of the sample was calculated based on the quantity of reactant and the total weight loss from the product over the temperature range of 50–800 °C, ignoring the slight weight loss of some samples beyond 800 °C attributable to the dehydroxylation of the silanol groups as C-S-H is transformed into wollastonite and SiO_2_ [33].

**Figure 6:**
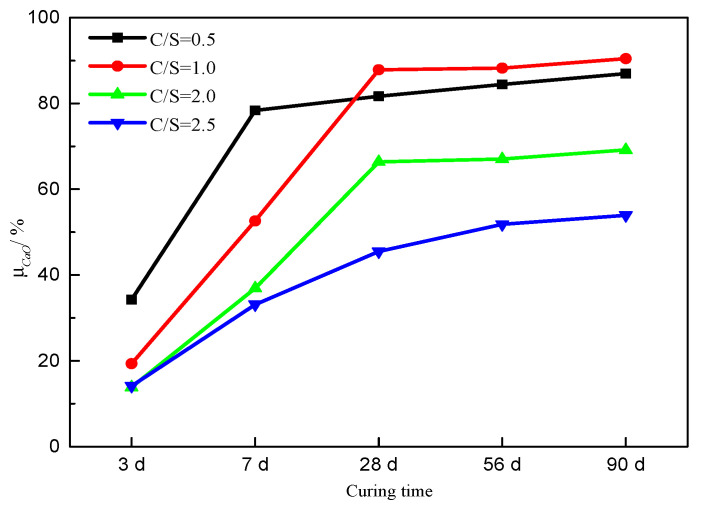
*μ_CaO_* values of the samples.

As shown in Figure 6, *μ_CaO_* decreased with increasing initial C/S molar ratio after curing times of 3 d and 7 d; it first increased and then decreased with an increase in the initial C/S molar ratio after curing times of 28 d, 56 d, and 90 d. It was maximized when the initial C/S molar ratio was 1.0 after curing times ≥ 28 d. Comparing Figure 5a and Figure 6, it can be seen that the trend in *μ_CaO_* with different initial C/S molar ratios after the same curing time was similar to that in weight loss from the C-S-H.

In the initial curing period (≤ 7 d), the greater the amount of silica fume added, the greater the value of *μ_CaO_* and the greater the weight loss due to C-S-H (as shown in Figure 5a). This indicated that it was predominately the content of silica fume that controlled the extent of the reaction and the amount of C-S-H formed. According to a previous study [34], in calcium hydroxide solution, silica first reacts with water to form a saturated solution of monosilicic acid, and then this monosilicic acid or its anion reacts with calcium hydroxide in the solution to form nuclei of calcium silicate hydrate [35]:

SiO_2_ (s) + 2H_2_O (l) = H_4_SiO_4_ (aq) (2)

H_4_SiO_4_ (aq) + *n*_1_Ca^2+^ (aq) + 2*n*_1_OH^−^ (aq) + (*n*_2_ − 2*n*_1_ − 1)H_2_O = *n*_1_CaO · SiO_2_ · *n*_2_H_2_O (s) (3)

In our reaction system, the calcium hydroxide solution was supersaturated at the beginning of the reaction. The solid calcium hydroxide continued to dissolve as Ca^2+^ in the solution was consumed. The greater the amount of silica fume added, the more of it that dissolved, and the higher the value of *μ_CaO_* and the weight loss due to C-S-H. Hence, the dissolution of silica controlled the kinetics of the overall reaction [35]. The values of *μ_CaO_* first increased and then decreased with increasing initial C/S molar ratio at the same curing time (≥28 d). This may be ascribed to the growth of C-S-H nuclei and better crystallinity with the extension of curing time, but it suppressed further dissolution of the silica.

SiO_2_ (s) + *n*_1_Ca^2+^ (aq) + 2*n*_1_OH^−^ + (*n*_2_ − 2*n*_1_)H_2_O = *n*_1_CaO · SiO_2_ · *n*_2_H_2_O (s) (4)

When the initial C/S molar ratio was 0.5, 1.0, or 2.0, the *μ_CaO_* values of the respective samples increased slightly with curing times ≥28 d. For an initial C/S molar ratio of 2.5, the *μ_CaO_* value of the sample cured for 56 d was not much different from that cured for 28 d but was noticeably higher for a sample cured for 90 d. In the later stage of curing (≥28 d), the *μ_CaO_* values of samples with initial C/S molar ratios of 0.5 and 1.0 were much higher than that for the sample with a C/S ratio of 2.5, consistent with the changes in weight loss due to C-S-H calculated from TGA data. In the case of longer curing time, too much or, in particular, too little silica fume will cause lower *μ_CaO_*. After curing for 90 d, the sample with an initial C/S molar ratio of 1.0 showed the highest *μ_CaO_* of 90.4%, as compared to 53.9% for the sample with a C/S molar ratio of 2.5.

Although C-S-H can be completely dissolved by a strong acid such as hydrochloric acid [36], it is difficult to separate the remaining solid phase from the solution medium [37]. In the present study, it proved difficult to determine the SiO_2_ contents in the C-S-H components of samples by chemical analysis and even more difficult to precisely determine their water contents [38]. Therefore, it was necessary to analyze the composition of C-S-H by other means.

### 3.3. XRD Analysis

**Figure 7:**
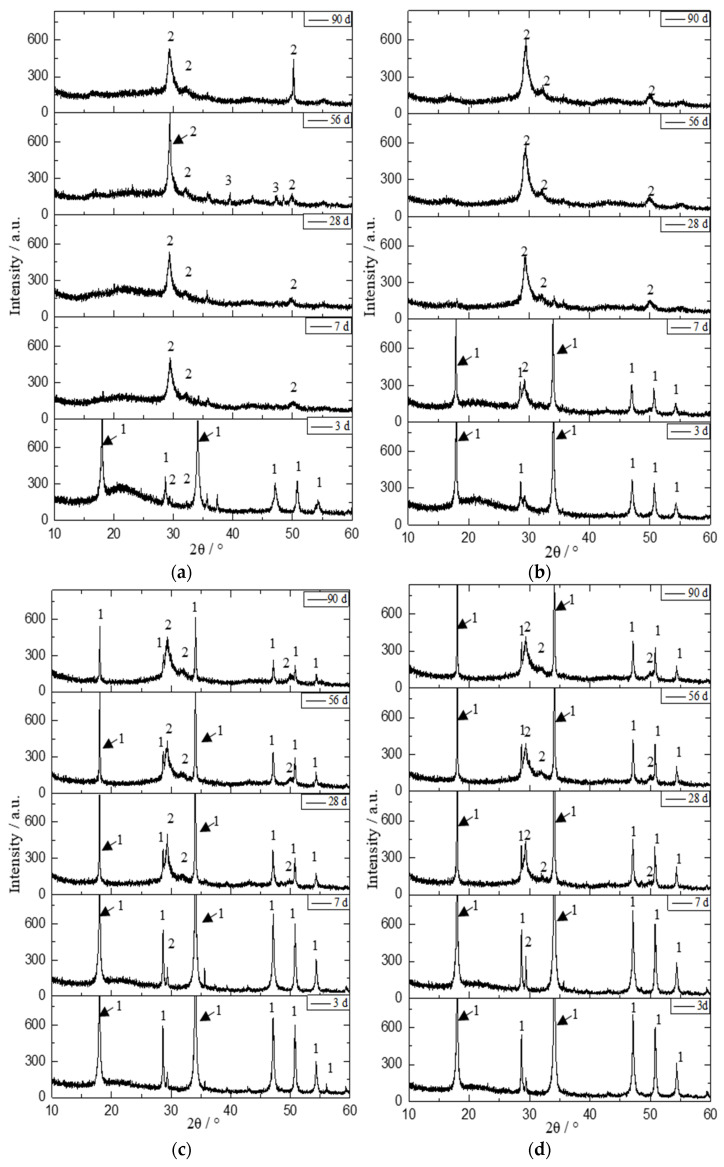
XRD patterns of samples with different curing times: (**a**) initial C/S molar ratio 0.5; (**b**) initial C/S molar ratio 1.0; (**c**) initial C/S molar ratio 2.0; and (**d**) initial C/S molar ratio 2.5. The assignment of the peaks is marked using the following abbreviations: 1—portlandite, 2—Ca_1.5_SiO_3.5_·*x*H_2_O, 3—CaCO_3_ (PDF#47-1743).

Figure 7 shows the XRD patterns of samples with different initial C/S molar ratios after different curing periods.

As shown in Figure 7a, the diffraction peaks of portlandite (Ca(OH)_2_, PDF#44-1481) were detected after a curing time of 3 d for the sample with an initial C/S molar ratio of 0.5. After a curing time of 7 d, the characteristic peaks of portlandite had disappeared, while three diffraction peaks due to C-S-H appeared at 2θ ≈ 29.4°, 32.1°, and 50.1°, corresponding to Ca_1.5_SiO_3.5_·*x*H_2_O (PDF#033-0306) [39,40]. Broad diffuse features in the 2θ range 15–30°, corresponding to the amorphous SiO_2_ phase as shown in Figure 1, persisted up to a curing time of 56 d. With the further increase in curing time, these broad diffuse features became less obvious. For the sample with an initial C/S molar ratio of 1.0, a difference was that there were still obvious diffraction peaks of portlandite and only one characteristic peak of Ca_1.5_SiO_3.5_·*x*H_2_O after a curing time of 7 d, compared with the pattern of the sample with an initial C/S molar ratio of 0.5. After a curing time of 28 d, the characteristic peaks of portlandite and the amorphous SiO_2_ phase had disappeared, and the three diffraction peaks of Ca_1.5_SiO_3.5_·*x*H_2_O had emerged. For the samples with initial C/S molar ratios of 2.0 and 2.5, a notable difference from the above results was that peaks due to portlandite were still present when the curing time was extended to 90 d. With increasing curing time, the diffraction peak intensity of portlandite decreased, consistent with the TGA results described above. For the samples with initial C/S molar ratios of 1.0–2.5, the characteristic peak of Ca_1.5_SiO_3.5_·*x*H_2_O was not obvious when the curing time was 3 d, which may be attributed to the amalgamation of the initially formed nuclei [35]. It can be seen from Figure 7 that the C-S-H consisted of Ca_1.5_SiO_3.5_·*x*H_2_O in the samples with different initial C/S molar ratios at curing times ≥ 28 d.

**Figure 8:**
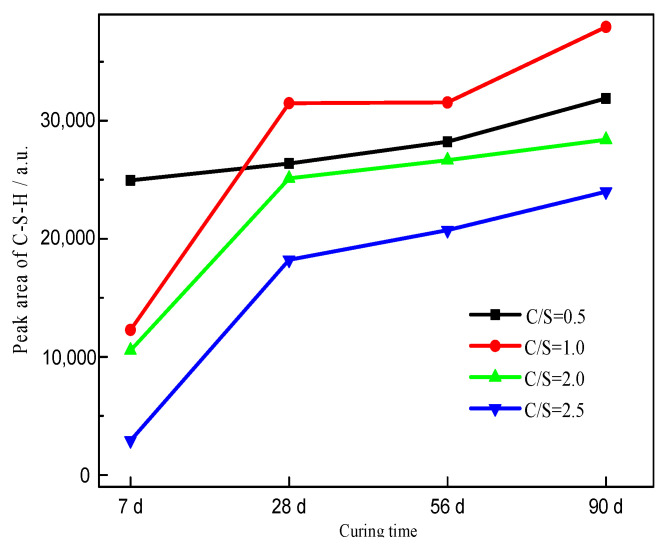
Peak areas of C-S-H in different samples.

The “Find Peaks” function in Jade 5.0 was used to determine the characteristic peaks in the pattern. The “Peak Search Report” function in the software was then applied to determine the height and area of each characteristic peak. The areas and heights of the peaks due to Ca_1.5_SiO_3.5_·*x*H_2_O in Figure 7 obtained from the “Peak Search Report” are listed in Table 3, and the peak area results for samples after curing times ≥ 7 d are shown in Figure 8. Clearly, the peak area of Ca_1.5_SiO_3.5_·*x*H_2_O increased with the extension of curing time for the same initial C/S molar ratio, indicative of the formation of more crystalline C-S-H [41]. The sample with an initial C/S molar ratio of 1.0 showed the highest peak area of C-S-H after curing times ≥ 28 d. This is consistent with the highest C-S-H content determined by TGA.

The value of *x* in Ca_1.5_SiO_3.5_·*x*H_2_O of all samples could be calculated in conjunction with the weight loss due to C-S-H in TGA and *μ_CaO_*. That is to say, after each curing time, the value of *x* in Ca_1.5_SiO_3.5_·*x*H_2_O varied with increasing initial C/S molar ratio in essentially the same way as the weight loss due to C-S-H. When the curing time was increased from 3 d to 90 d, the values of *x* were 1.8–3.2 for the samples with initial C/S molar ratios of 0.5–2.5. The different values of *x* may be related to defects in the C-S-H structure [42]. For curing times of 3 d to 90 d, the possible reaction between calcium hydroxide and silica fume may be expressed as follows:

SiO_2_ (s) + 1.5Ca^2+^ (aq) + 3OH^−^ (aq) + (0.3–1.7)H_2_O = 1.5CaO·SiO_2_·(1.8–3.2)H_2_O (s) (5)

### 3.4. ^29^Si MAS-NMR Analysis

**Figure 9:**
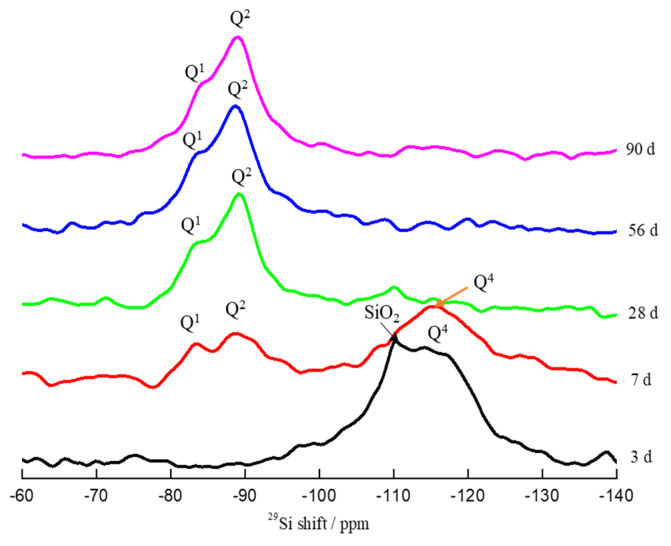
^29^Si MAS-NMR spectra of samples with initial C/S molar ratio of 1.0 after different curing times.

Samples with an initial C/S ratio of 1.0 were analyzed after curing times by ^29^Si MAS-NMR. In the ^29^Si MAS-NMR spectra (Figure 9), the tetrahedral coordination is expressed as Q^1^, Q^2^, and Q^4^, based on the chemical shifts (ppm) of silicon atoms bonded to *n* bridging oxygen atoms. Q^1^ denotes a chain-end tetrahedron, Q^2^ denotes a chain intermediate tetrahedron (silica tetrahedra coordinated to a calcium ion), and Q^4^ denotes a three-dimensional network structure formed from four silica tetrahedra [43]. With the increase in curing time, SiO_2_ and Q^4^ (silica gel) disappeared (curing time ≥ 28 d), while Q^1^ and Q^2^ appeared (curing time ≥ 7 d). The Q^2^/Q^1^ ratio reflects the degree of polymerization of solid C-S-H; that is, the higher the value, the longer the linear silicate chains [44]. The relative proportions of Q^1^ and Q^2^ were determined by deconvolution of the spectra using the software Peakfit v4.12. For samples cured for 7 d, 28 d, 56 d, and 90 d, the deduced Q^2^/Q^1^ ratios were 2.29, 4.10, 4.24, and 4.28, corresponding to the samples with curing times of 7 d, 28 d, 56 d, and 90 d, respectively. Thus, the degree of polymerization of C-S-H increased with increasing curing time.

### 3.5. Analysis of the Formation Mechanism of C-S-H

**Figure 10:**
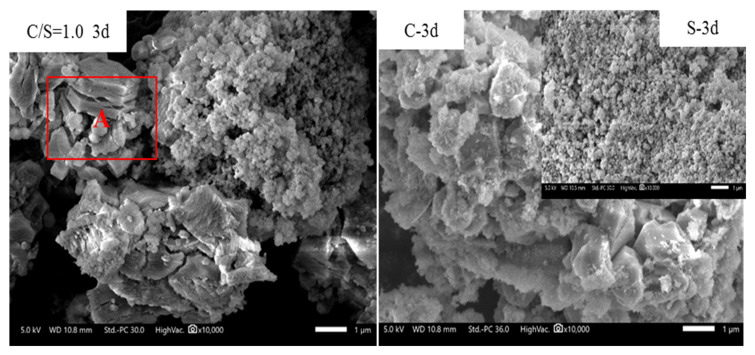

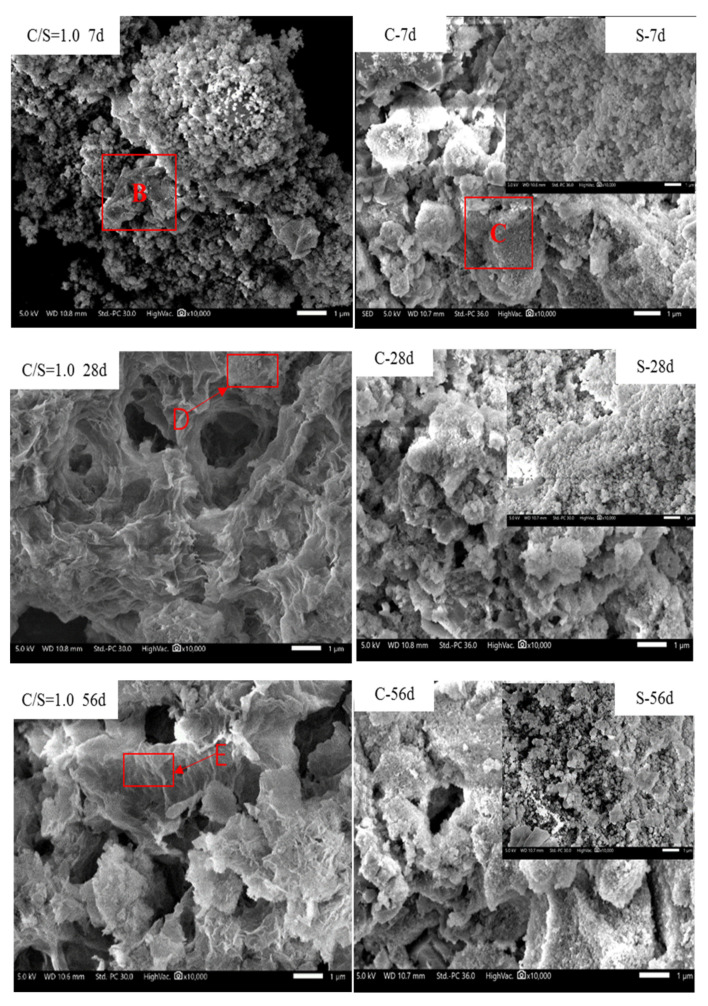

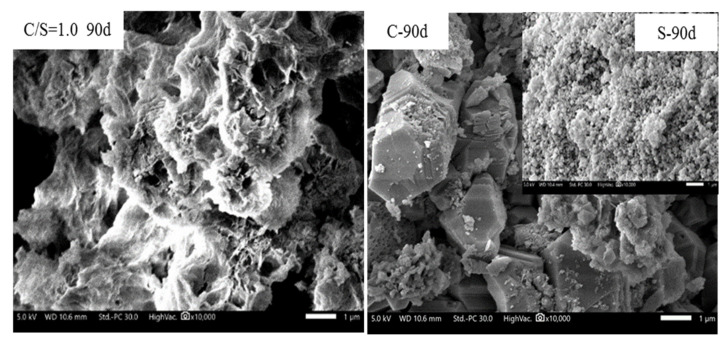
Microstructures of samples with an initial C/S molar ratio of 1.0.

The microstructures of samples with different initial C/S molar ratios after different curing times were observed by SEM. For each initial C/S molar ratio, the changes in the microstructural characteristics of the samples were similar with the extension of curing time. Therefore, the sample with an initial C/S molar ratio of 1.0 is taken as an example to analyze the microstructural characteristics of the samples after different curing times, as shown in Figure 10.

In order to better analyze the C-S-H formation process, control samples marked with C-curing time and S-curing time were prepared, images of which are also shown in Figure 10. The preparation process of the samples marked with C-curing time was similar to that of the samples previously studied, except that silica fume was not added in the preparation process. The samples marked with S-curing time were prepared by a similar method to those with different initial C/S molar ratios but omitting calcium oxide from the reactants.

When the curing time was 3 d or 7 d, spherical particles similar to those in the samples marked S-3 d to S-90 d were still evident, whereas the morphology of the calcium hydroxide particles (similar to that reported previously [45]) in the samples with different initial C/S molar ratios was obviously different in the samples marked C-3 d to C-90 d. In the calcium hydroxide structure of the former samples, there were not only holes, as in the region C marked in red in Figure 10, but also another structural fracture phenomenon, as shown in regions A and B, indicating that the addition of silica fume led to the cracking of calcium hydroxide into smaller fragments, resulting in a larger specific surface area and hence higher activity.

**Figure 11:**
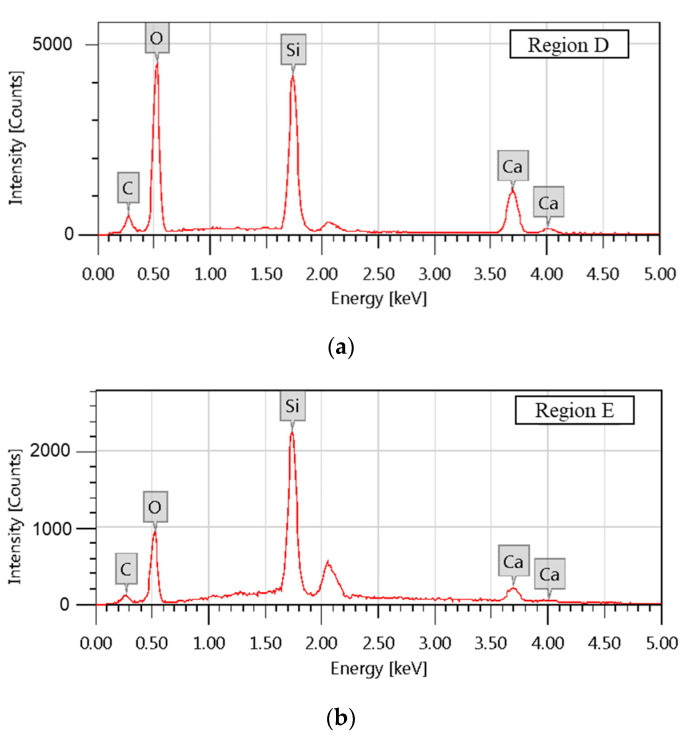
EDS results at region D (**a**) and at region E (**b**).

The typical crystal structure of C-S-H could be observed when the curing time was ≥28 d. Although the overall shape was a leaf-like C-S-H structure [46], which was a little different from those described before [47], it is interesting to note some inhomogeneity therein. Two regions with obviously distinct characteristics are marked D and E in Figure 10. Combined with the energy-dispersive spectroscopy results shown in Figure 11 and Table 4, it is evident that the morphology of region D is akin to velvet and is composed of Si-rich C-S-H, whereas region E is more like crumpled sheets and is composed of Ca-rich C-S-H. No particles of calcium hydroxide or substrates thereof can be discerned in these images. We propose a formation mechanism of C-S-H based on the above analyses.

**Figure 12:**
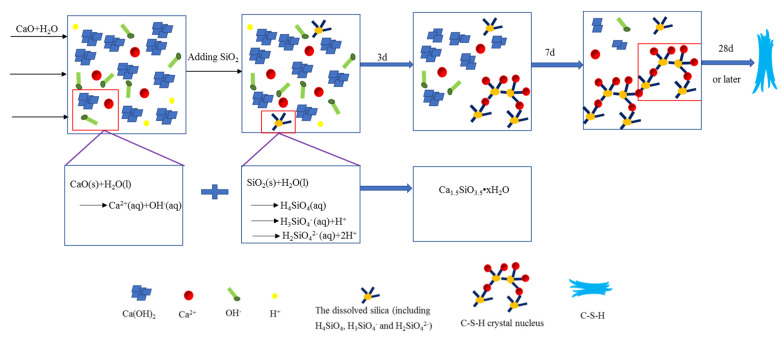
Schematic diagram of the formation of C-S-H.

First, the raw material calcium oxide reacts with ultrapure water to form slightly soluble calcium hydroxide, which exists in the form of Ca^2+^ and OH^-^ ions in the solution. On adding silica fume to the solution, it reacts with water to form H_4_SiO_4_ [34]. As the calcium hydroxide particles fracture, their contact area with water and silicic acid increases; the Ca^2+^ dissolved in water reacts with H_3_SiO_4_^−^ and H_2_SiO_4_^2−^ dissociated from H_4_SiO_4_ (aq) [48] to form C-S-H crystal nuclei. These do not adhere to the surface of calcium hydroxide; rather, ectopic nucleation occurs. With the progress of the reaction, Ca^2+^ is continuously precipitated and continues to react with the dissolved silica and the amount of C-S-H crystal nuclei increases. Under the influence of thermal motion, crystal nuclei will attract and collide with one another. The crystal nuclei eventually agglomerate and grow and continue to absorb surrounding small particles, constituting a “polymerization reaction”. This can also explain the densification of leaf-like C-S-H after curing times of 56 d and 90 d compared with 28 d, as indicated by the XRD results. Finally, the system reaches an equilibrium state. A schematic diagram of the ectopic nucleation–polymerization reaction process involved in the formation of C-S-H is shown in Figure 12.

## 4. Conclusions

The effects of different initial C/S molar ratios and curing times on the formation of C-S-H have been studied. The following conclusions can be drawn:

For samples with the same C/S molar ratio, the weight loss from C-S-H and the *μ_CaO_* value increased with increasing curing time from 3 d to 90 d, while the weight loss from calcium hydroxide decreased. The maximum values of the weight loss due to C-S-H and *μ_CaO_* were reached when the initial C/S molar ratio was 1.0. The amount of C-S-H and the *μ_CaO_* value were both maximized (13.5% and 90.4%, respectively) when the sample with an initial C/S molar ratio of 1.0 was cured for 90 d. XRD analysis revealed the crystal type of C-S-H in the samples to be Ca_1.5_SiO_3.5_·xH_2_O. The increase in curing time was beneficial to crystal growth. The value of x in Ca_1.5_SiO_3.5_·xH_2_O was calculated from the TGA results and varied in the range of 1.8–3.2. ^29^Si MAS-NMR spectra of the samples with an initial C/S ratio of 1.0 after different curing times have shown that Q^1^ and Q^2^ appeared after a curing time of 7 d. The degree of polymerization of C-S-H increased (manifested in an increase in the Q^2^/Q^1^ ratio from 2.29 to 4.28) with an increase in curing time. A leaf-like C-S-H structure was observed in SEM images of the samples with different initial C/S molar ratios after curing times ≥ 28 d. Si-rich C-S-H and Ca-rich C-S-H structures were observed in the same image. An ectopic nucleation–polymerization reaction for the formation mechanism of C-S-H is proposed.

It is hoped that this work may expedite the hydration and use of fly-ash-based cementitious materials through the optimization of their components in terms of stoichiometry and phase composition. Nevertheless, it remains necessary to further study the influences of other prominent substances in fly ash, such as Al_2_O_3_, MgO, and Fe_2_O_3_, on the formation of C-S-H.

## Figures and Tables

**Table 1:** Chemical composition of silica fume (wt%).

Component	SiO_2_	Al_2_O_3_	K_2_O	CaO	Fe_2_O_3_	MgO	Na_2_O	Loss
Content	92.82	0.49	0. 53	0.59	0.29	0.49	0.42	4.37

**Table 2:** Characteristic parameters of thermal weight loss.

Initial C/S Molar Ratio	Weight Loss Due to C-S-H/%					Weight Loss Due to C−H/%					Weight Loss Due to CaCO_3_/%				
	3 d	7 d	28 d	56 d	90 d	3 d	7 d	28 d	56 d	90 d	3 d	7 d	28 d	56 d	90 d
0.5	3.1	7.9	8.1	11.6	12.5	4.8	1.1	0.8	0.6	0.4	1.8	1.7	1.6	1.6	1.5
1.0	2.8	6.7	12.2	13.0	13.5	9.3	5.1	0.8	0.8	0.4	1.8	1.8	1.6	1.6	1.8
2.0	2.1	5.4	9.6	11.4	11.7	12.7	8.7	4.2	3.5	2.3	3.1	3.3	2.8	4.1	6.0
2.5	2.2	5.0	7.6	8.1	8.5	13.9	9.8	8.0	6.8	6.4	3.0	3.7	3.0	3.3	3.4

**Table 3:** XRD peaks of C-S-H.

Initial C/S Molar Ratio	Mineral Composition	2θ/°	Area				Height			
			7 d	28 d	56 d	90 d	7 d	28 d	56 d	90 d
0.5	Ca_1.5_SiO_3.5_·*x*H_2_O	29.4	20,254	19,548	25,382	20,727	278	285	616	276
		32.1	1520	2963	1542	3161	31	48	47	50
		50.1	3183	3854	1297	9449	35	53	22	338
	Total		24,957	26,365	28,221	31,894	344	386	685	664
1.0	Ca_1.5_SiO_3.5_·*x*H_2_O	29.4	12,283	22,292	23,702	29,711	172	289	212	390
		32.1	—	3756	3009	2999	—	47	154	43
		50.1	—	5435	4835	5222	—	54	90	58
	Total		12,283	31,483	31,546	37,932	172	390	456	491
2.0	Ca_1.5_SiO_3.5_·*x*H_2_O	29.4	10,559	20,048	18,915	21,723	123	319	233	248
		32.1	—	2192	2847	2440	—	32	40	37
		50.1	—	2880	4898	4231	—	23	40	46
	Total		10,559	25,120	26,660	28,394	123	374	313	331
2.5	Ca_1.5_SiO_3.5_·*x*H_2_O	29.4	2923	15,238	17,315	17,566	219	209	238	228
		32.1	—	2964	2585	2608	—	31	37	36
		50.1	—	20	831	3820	—	10	16	83
	Total		2923	18,222	20,731	23,994	219	250	291	347

**Table 4:** EDS results for the marked regions in Figure 10.

Region	Atomic Composition /%			
	C	O	Si	Ca
D	8.86 ± 0.08	59.80 ± 0.28	17.95 ± 0.12	13.39 ± 0.13
E	1.77 ± 0.33	18.65 ± 0.20	33.45 ± 0.32	46.13 ± 1.33

## Data Availability

Not applicable.
